# The Dallas Meeting

**Published:** 1902-06

**Authors:** 


### THE DALLAS MEETING.

The thirty-fourth annual meeting of the State Medical Association of Texas was held at Dallas, May 6 to 9, inclusive. There was a good attendance, and a splendid program was carried out. Elsewhere will be found an editorial by our junior, Dr. Russ, on the differences that unfortunately arose over the adoption of a new constitution. On account of press of matter and limited space, I give here and now only a few items of the meeting, and must omit much that I would like to publish. There were many pleasant social features of the occasion which were greatly enjoyed. The papers read were mostly valuable, and will appear in the volume of transactions. The Association is not making much headway in membership, the roll showing, according to the Secretary's report, 373 members. Cash on hand, $1,628.35. This amount was increased to something over $2,000 by this meeting. A large number of new members was added to the roll.

MEMORANDA OF THE MEETING—OFFICERS FOR THE YEAR.

*President,* Dr. S. E. Red, Houston, Texas.

*First Vice-President,* Dr. J. E. Thompson, Galveston.

*Second Vice-President,* Dr. J. E. Gilcreest, Gainesville, Texas.

*Third Vice-President,* Dr. H. K. Leake, Dallas, Texas.

*Trustees,* Dr. F. D. Thompson, Fort Worth; Dr. J. D. Osborn, Cleburne; Dr. W. Shropshire, Yoakum; Dr. J. B. Shelmire, Dallas; Dr. Jos. Becton, Greenville.

*Judicial Council,* Dr. J. S. Lankford, San Antonio; Dr. C. C. Gibney, Granger; Dr. H. A. Barr, Beaumont, and Dr. J. O. McReynolds, Dallas.

*Orator,* Dr. M. L. Graves, San Antonio.

*Delegate to American Medical Association,* Dr. H. A. West; Alternate, Dr. T. J. Wilson.

*Time and place of 1903 meeting,* San Antonio, fourth Tuesday in April.

Dr. R. H. Harrison of Columbus, Ex-President, was made an honorary member.

*Committee on State Board of Health,* Dr. F. E. Daniel, Austin, Chairman; Dr. Frank Paschal, San Antonio; Dr. Bacon Saunders, Fort Worth; Dr. P. C. Coleman, Colorado City; Dr. W. R. Blailock, McGregor.

OFFICERS OF SECTIONS.

*General Medicine,* Dr. W. C. Blalock, of Kosse, Chairman; Dr. J. T. Moore, of Galveston, Secretary.

*Obstetrics and Diseases of Children,* Dr. J. C. Anderson, of Granger, Chairman; Dr. C. E. Cantrill, of Greenville, Secretary.

*Surgery,* Dr. W. Keiller, of Galveston, Chairman; Dr. J. P. Gunby, of Sherman, Secretary.

*Medical Jurisprudence,* Dr. J. R. Nichols, of Terrell, Chairman; Dr. R. B. Sellers, of San Antonio, Secretary.

*State Medicine,* Dr. Frank Paschal, of San Antonio, Chairman; Dr. H. W. Crouse, of Victoria, Secretary.

*Gynecology,* Dr. J. Smoot, of Dallas, Chairman; Dr. H. Taylor, of Marshall, Secretary.

*Ophthalmology,* Dr. Henry C. Haden, of Galveston, Chairman; Dr. W. A. Harper, of Austin, Secretary.

*Dermatology,* Dr. W. B. Russ, of San Antonio, Chairman; Dr. H. B. Decherd, of Galveston, Secretary.

*Pathology,* Dr. A. J. Smith, of Galveston, Chairman; Dr. J. M. Frazier, of Belton, Secretary.

---

CONSUMPTION IN TEXAS.—Dr. W. S. Carter, of Galveston, read the report of the Committee on "Uniform Action for the Prevention of Tuberculosis in Texas." Among other recommendations made were these: That local boards of health declare the disease communicable and seek to have the cases isolated; to encourage the passage and enforcement of anti-expectoration ordinances because of their educational value; to continue the examination of dairy herds for tuberculosis; to ask the State to establish a bacteriological laboratory to conduct examinations for tuberculosis at public expense; to ask the State to establish and support a hospital for indigent consumptives; to disseminate information as to the disposition of sputum.

Speaking to this report, Dr. Frank Paschal, of San Antonio, health officer of that city and a delegate appointed by Governor Sayers to represent the State of Texas at the tuberculosis congress to be held in New York, delivered an address which excited much interest. He said he would give the Association some statistics compiled from the mortuary records of the City of San Antonio for the past thirty years, the accuracy of the figures having been assured by a triple examination. Tuberculosis, he said, has spread rapidly in this State, especially in the places where consumptives have congregated. In 1870 there were eighteen deaths in San Antonio from tuberculosis, being 1.4 persons to every 1,000 population of the city. At that time San Antonio was isolated; there was no railroad connection with the city and consumptives could not well go there. In 1880 conditions had changed; railroad connection was established, and the city was widely advertised as a health resort. The result was that in 1880 there were 3.3 deaths from tuberculosis to every 1,000 population, in 1890, 4 and a fraction, in 1900, 6, an increase of 600 per cent. in thirty years. It might be said that this increase was due to new comers afflicted with the disease. The statistics do not account for it in that way. Of the total number who died from tuberculosis in San Antonio during the thirty years, forty and a fraction per cent. had lived in the city one year and under. Thirteen per cent. of them had lived in San Antonio ten years and over. The duration of tuberculosis is from two to seven years, rarely as much as ten years. It is therefore evident that those who had lived in San Antonio ten years or over contracted the disease there. In 1900, 26 per cent. of those dying in San Antonio from tuberculosis had lived there ten years or over. In 1890 there were twenty-three deaths from tuberculosis of persons who had resided in San Antonio ten years or over, and in 1900 eighty such deaths, an increase of 300 per cent.

"You may ask why should such figures be given out concerning a city which is a health resort," Dr. Paschal continued. "It has been suggested to me that they might be injurious to commerce, but the lives of our people are of more value than commerce. It behooves every man to speak out, although commerce may be imperiled. I have been told that if I let these facts get out it would injure the city, and I replied, 'Gentlemen, if you are willing to sacrifice the lives of your wives and children and of your friends for the sake of commerce, then God save the mark. I say let the truth be known, that we may have legislation to stop the spread of the disease in San Antonio and throughout Western Texas and the State generally.'

"One-fifth of the entire number of deaths in San Antonio last year died in the city hospital, and were absolute paupers. Of that one-fifth, I don't suppose one-tenth of them lived in Texas. They came to San Antonio and said, 'You must take care of us; if you don't take us in the hospitals we will sit down on your steps or in the streets.' We are obliged to take them in and care for them until they die or go away to scatter the seeds of disease. It is not right that we should be taxed that way. The State should build an institution to care for these indigents and keep them isolated. When the State builds an institution for indigent consumptives it will stop this promiscuous immigration. The very day that they learn that they will become wards of the State and be restrained, they will quit coming, for they have a horror of being huddled together. The probability is that if the State establishes such an institution we will have to care for the indigent consumptives of Texas only, and the disease will decline when this reckless tuberculosis immigration is arrested. Not till then will legislation stop the spread of the disease. We must stop the source of the disease supply, which is the indigent consumptives. I don't want to put a pebble in the way of any man's recovering his health, but it is our duty to protect our people from disease. It is not difficult to control the non-indigent consumptives. It is the indigent and ignorant that give trouble."

---

GOVERNOR SAYERS NOW FAVORS A STATE BOARD OF HEALTH.

At this juncture Governor Sayers was escorted to the hall and to the platform, being received with applause. In introducing the distinguished guest, Dr. J. T. Wilson, of Sherman, said: "He is a man who is broad and liberal enough to take in the whole political situation, not only of Texas, but the United States as well; also to take in all the sciences and every other interest of the State. He is the friend of the Medical Association. He has done more for medicine and the charitable institutions of the State than any other man I know. His message was the cause of the enactment of the law by which the medical practice of the State is now governed."

These remarks were greeted with applause, and the Governor proceeded to address the Association. In acknowledging the compliment paid him by Dr. Wilson, the Governor said it was not altogether deserved, for had he not had the assistance of Dr. Wilson and others of the medical profession, the measure would not have been enacted. He said that he was not a physician, but the son of a physician, who from the time he graduated at the University of Pennsylvania until his seventy-fifth year, gave his entire life to the practice of medicine. Governor Sayers said he knew from observation that the life of the honest, true, conscientious physician is one of labor and sacrifice beyond the knowledge of the world; he is a man who letteth not the right hand know what the left hand doeth, and who gives liberally of his means and services to the poor.

Governor Sayers then told how he had become impressed with the belief that the practice of medicine in Texas was not properly regulated; how he had asked Dr. Wilson to prepare a bill, and how the present law had been enacted. Continuing, he said that in his judgment as a layman, it was strongly necessary to enact another measure. The health department is controlled by a single man, although the statutes impose duties in important matters upon the Governor.

"There ought to be a thorough reorganization," Governor Sayers continued. "The Health Department, instead of being confined to duties of a general character at Austin, ought to be able, through regularly appointed boards, under authority of law, to ascertain conditions in every village, town, city and county throughout the State. There is now no law providing for such boards. The legislation which we need ought not to be cumbersome. If there are too many boards or too many members of the boards; if the plan is expensive or otherwise cumbersome, the law will never be executed.

"I know you will not agree with me, but I am going to give you my experience with the Health Department. There has been much criticism in regard to quarantine. That has been due to a want of thorough knowledge of the circumstances in each case. During the time I have been Governor, the relations between the Health Departments of Texas and Louisiana, we found that we could not at all times rely with entire confidence upon the reports from Louisiana, and furthermore, we found that our railroad systems, instead of observing the agreements reached with the Health Department of Texas in regard to certain kinds of traffic, were apt to violate them. Therefore, in the interest of the health of the people of Texas, we were required to establish quarantine where apparently there was no necessity for it. Last year and during a part of this year we succeeded in having such a written agreement with the railroads that we were able to be more lax in quarantine regulations, while protecting the health of the people of Texas.

"Texas is a large State and there are many points of entrance. We found that if a quarantine were established against Louisiana, it might be observed by, say, the Southern Pacific, passing through Beaumont, while forbidden goods were forwarded via Shreveport and the Texas and Pacific, or via Vicksburg and Little Rock. No matter who is State Health Officer, no matter whether the next Legislature and the next Governor in their wisdom may see proper to establish a board which shall determine whether quarantine shall be established, you will find that there will be friction and things will be done which on their face may seem unnecessary. The State Health Officer or the Governor can not afford to go into print with all the facts at such times. I state these things, not in criticism, but that you may understand the difficulties surrounding the Health Department as at present constituted."

To emphasize the necessity of having a quarantine law which is simple and not cumbersome, Governor Sayers cited the fact that the United States quarantine law was so cumbersome that it fell of its own weight and although it had not been repealed it has not been enforced for fifteen years. In this connection, Governor Sayers paid a tribute to the fine ability of Dr. Wyman, Surgeon General of the United States Marine Hospital Corps, in charge of the national quarantine, and testified to the satisfactory and pleasant relations between Dr. Wyman and the Health Department of Texas. He also paid a glowing tribute to the worth of the services rendered by the two health officers who have served under his administration—Dr. Blunt, and the present incumbent, Dr. Tabor.

With respect to the drafting of a new quarantine law, the Governor admonished the doctors that they would find it a much more difficult task than they anticipated. If they were going to intrust it to a committee, the committee should get to work early, draft the bill and furnish every physician a copy for inspection and criticism. Then, with all the criticisms before them, the committee should draft a new bill and have it on the desks of every member of the Legislature when the lawmaking body meets in next January. The Governor spoke in most complimentary terms of the physicians of Texas, saying that no more important work confronted them than to protect the State from the invasion of disease and to prevent disease within the State.—*Dallas News* Report of Meeting.

---

The Section on State Medicine and Public Hygiene was taken up. Dr. W. S. Carter, Chairman of the Section, read his address, in which he commented on the great progress made during recent years in preventive medicine.

Dr. F. E. Daniel, of Austin, read a paper on "The Paramount Duty Texas Owes Her People." He prefaced the reading of the paper by saying he had been gratified by hearing the Governor express himself during the afternoon practically in line with his paper. He said that it was not the Governor's fault that the medical practitioner bill had been emasculated by the Legislature; at the same time, but without criticising the Governor, he regretted that the Governor had not expressed himself when the several committees of the State Medical Association called on him during the sitting of the Legislature, as fully and as strongly as he had done at this meeting. Referring to his paper criticising the quarantine system of Texas, he said he desired it understood that he was criticising the system and the law, and not the distinguished gentlemen who had administered the law. In his paper he criticised the Health Department severely, and said it was remarkable that the efforts of the State Medical Association for twenty-five years had failed to convince the Governors and legislators of Texas that the health system is a disgrace to the State.

The paper was discussed by Dr. Taylor Hudson and Dr. Frank Paschal aprovingly. Dr. Hudson said he thought the paper should be placed in the hands of every family in Texas. Dr. Páschal said the prospects for proper health legislation are better now than ever before. A year ago, he said, he had been quoted in a newspaper as saying that the Governor advocated a State Board of Health. The next year the Governor disclaimed the statement and was entirely correct. But now the Governor has told the State Medical Association that he favors a State Board of Health, which Dr. Paschal said was very encouraging.

Dr. M. M. Smith, of Austin, further discussed the paper and was followed by Dr. George Tabor, State Health Officer, who said he had enforced the laws as he found them, he could not go beyond the laws, except by request. He said he had recently requested the railway companies to improve the sanitary conditions of their passenger cars, and was happy to say seventy-five per cent. of them had granted his request. There would always be complaints concerning the coast quarantine on the part of commercial interests, he said. The regulations, however, are milder now than ever before. "The coast quarantine regulations in Texas now are as lax as they can be to comply with the United States Marine Hospital regulations," said Dr. Tabor. "If we were to make the restrictions lighter the Marine Hospital Service would supersede us in the Texas coast quarantine." The Department cannot under the law and with the limited appropriations inaugurate any further work such as has been suggested and which should be done.

Dr. M. M. Smith moved that the Association authorize the publication and distribution of Dr. F. E. Daniel's paper on "The Paramount Duty of the State of Texas," Dr. W. S. Carter's paper on "State Medicine," dealing with the prevention of contagious, infectious and communicable diseases; also to publish and distribute a paper advising the public of the danger of tuberculosis and how to prevent its spread.

Dr. West suggested that the amount to be expended should be stated, and he offered an amendment to make it $50. Drs. Carter and Smith suggested $200. Dr. Miller, Treasurer of the Association, was called upon to say how much the Association could stand. He at first declined to do so, merely stating that there was $2000 in the treasury, but when Dr. J. E. Thompson moved to appropriate $500, Dr. Miller said the Association would go broke if it went to throwing money at birds. He said he had worked hard to get the Associaton in a good financial condition and he would regret to see its treasury emptied.

The Smith motion was finally passed, carrying an appropriation of $200.—*Dallas News* Report of Meeting.

[These papers will be printed in pamphlet form by the Committee on State Board of Health.—Ed.]

